# Integrated Targeted and Suspect Screening Workflow for Identifying PFAS of Concern in Urban-Impacted Serbian Rivers

**DOI:** 10.3390/toxics14010078

**Published:** 2026-01-14

**Authors:** Igor Antić, Maja Buljovčić, Richard E. Cochran, Jelena Živančev, Marta Llorca, Marinella Farré, Dušan Rakić, Ralf Tautenhahn, Nataša Đurišić-Mladenović

**Affiliations:** 1 University of Novi Sad, Faculty of Technology Novi Sad, Bulevar cara Lazara 1, 21000 Novi Sad, Serbia; antic@tf.uns.ac.rs (I.A.); majab@tf.uns.ac.rs (M.B.); jelena.zivancev@tf.uns.ac.rs (J.Ž.); rakic.11.20.d@uns.ac.rs (D.R.); 2 Thermo Fisher Scientific, Bannockburn, IL 60015, USA; richard.cochran@thermofisher.com; 3 Environmental and Water Chemistry for Human Health (ONHEALTH), Institute of Environmental Assessment and Water Research (IDAEA-CSIC), C/Jordi Girona 18–26, 08034 Barcelona, Spain; marta.llorca@idaea.csic.es (M.L.); mfuqam@cid.csic.es (M.F.); 4 Thermo Fisher Scientific, San Jose, CA 95134, USA; ralf.tautenhahn@thermofisher.com

**Keywords:** per- and polyfluoroalkyl substances, suspect screening, targeted analysis, high-resolution mass spectrometry (HRMS), risk assessment, risk-based prioritization

## Abstract

This study presents the first comprehensive assessment of per- and polyfluoroalkyl substances (PFAS) in surface waters of northern Serbia (Middle Danube region), combining targeted analysis of 25 PFAS with high-resolution mass spectrometry suspect screening (SSA) at 12 settlement-adjacent sites on major rivers and part of the Danube–Tisa–Danube (DTD) canal network. The sum of 10 quantified PFAS showed pronounced spatial variability: the Great Bačka Canal (GBC) exhibited the highest mean and maximum values (18.4 ng/L and 52.6 ng/L, respectively); the Danube averaged 9.05 ng/L (2.92–22.2 ng/L); the Tisa averaged 10.5 ng/L (4.53–16.5 ng/L); and the Sava and Tamiš exhibited the lowest means (~5.4 ng/L each). In total, 19 of 24 sites exceeded the proposed EU group Environmental Quality Standard (EQS) of 4.4 ng/L, expressed as PFOA-equivalents, with exceedances of 5.4–20.2 ng/L; PFOS exceeded the 0.65 ng/L inland surface water annual average (AA) EQS in 17 samples. SSA expanded coverage beyond targets, revealing ultra-/short-chain PFAS and replacements, with TFA as the most abundant (337–1165 ng/L; mean 513 ng/L) and notable maxima for PFPrA (51.3 ng/L), ADONA (24.9 ng/L), and TFMS (11.2 ng/L). Compared with European freshwaters, the maximum obtained here lies in the lower-mid part of the reported range, consistent with short-chain perfluoroalkyl carboxylic acids (PFCA) dominance and diffuse-source influences.

## 1. Introduction

Per- and polyfluoroalkyl substances (PFAS) are among the most concerning classes of contaminants of emerging concern (CECs) due to their extreme environmental persistence, bioaccumulation, and toxicity. They are characterized by the presence of at least one fully fluorinated carbon atom (–CF_2_– or –CF_3_) without hydrogen or halogen substitution [1,2]. Owing to their chemical and thermal stability, surfactant-like properties, and amphiphilic nature (hydrophobic and lipophobic properties), PFAS have been widely used in industrial and consumer applications, such as non-stick coatings, lubricants, firefighting foams, and electronics. However, these same properties have resulted in their global distribution and long-term persistence in the environment [3]. Perfluorooctanesulfonic acid (PFOS), perfluorooctanoic acid (PFOA), perfluorohexanesulfonic acid (PFHxS), and perfluorononanoic acid (PFNA) are listed under the Stockholm Convention on Persistent Organic Pollutants due to their adverse health and ecological effects [4]. Hydrogen-substituted perfluoroalkyl carboxylic acids (H–PFCAs) are PFAS alternatives that have recently emerged in the environment. As a consequence of increased regulation of long-chain PFAS, the industry has shifted its production to shorter-chain length and replacement chemistries, such as perfluoro-2-hydroxypropanoic acid (HFPO-DA), a perfluoro ether carboxylic acid [5].

Across Europe, PFAS have been widely reported in surface water, sediment, and biota [6,7,8,9]. However, data for the Danube River Basin (DRB), particularly in Serbia, remain scarce. Only a few research-driven studies have examined PFAS contamination in Serbia. Beškoski et al. [10] analyzed a single PFAS compound in sediment from a Danube-connected canal, while Buljovčić et al. [11] performed targeted analysis of 11 PFAS in surface waters of the Danube River at two monitoring sites. At the regional level, Ng et al. [12] conducted a basin-wide PFAS investigation within the Joint Danube Survey 4 (JDS4) [13], applying combined targeted and suspect screening to river water, wastewater, groundwater, and biota. Within Serbia, only three surface water sampling locations were included, which were the only Serbian sites in the survey. Across the full JDS4 dataset, 18 out of the 82 PFAS investigated were identified as being of potential environmental concern, demonstrating the widespread and transboundary nature of PFAS contamination and the need for harmonized monitoring in Southeast Europe.

The advancement of high-resolution mass spectrometry (HRMS) and data-processing software has significantly expanded the possibilities for SSA and non-targeted analysis (NTA), enabling the detection of previously unknown contaminants in complex environmental matrices such as water, soil, plants, and even human tissue [14]. High-resolution techniques such as LC–HRMS and GC–HRMS are increasingly used for SSA and NTA, enabling tentative identification of unknown or emerging PFAS based on accurate mass, retention time, and fragmentation patterns [15]. In SSA workflows, fragmentation patterns used for compound identification can be obtained experimentally, through MS/MS acquisition (e.g., data-dependent acquisition (DDA) and data-independent acquisition (DIA)), or predicted in silico using computational tools such as MetFrag, CFM-ID, or MS-FINDER. While experimental spectra provide high-confidence structural confirmation, in silico fragmentation plays a crucial role in annotating compounds for which no reference standards or spectral library entries are available. SSA of PFAS requires HRMS platforms with high mass accuracy and sensitivity to detect both precursor and fragment ions, typically produced via higher-energy collisional dissociation (HCD). The choice of acquisition mode (DDA, DIA, or all ion fragmentation (AIF)) depends on the instrument type (e.g., Orbitrap, TOF, QTOF) and the scientific objective, each offering distinct trade-offs in selectivity and retrospective applicability. Due to the limited availability of PFAS standards, comprehensive spectral databases such as mzCloud, MassBank, MetFrag, CFM-ID, and the EFS HRAM database are essential. Finally, confidence level frameworks [16,17] are widely used to rank compound annotations [18], although inconsistencies between laboratories persist due to variations in instrument resolution, database coverage, and workflow design.

The presence of certain PFAS compounds in food and drinking water is regulated at both EU and global levels, with limits set for PFOS, PFOA, PFNA, PFHxS, and related compounds in line with Commission Recommendation (EU) 2022/1431, the EU Drinking Water Directive, and WHO recommendations [19,20]. For surface waters, the EU Water Framework Directive [21] sets stringent environmental quality standards for PFOS and its derivatives, with recent proposals introducing a cumulative threshold for 25 PFAS (including trifluoroacetic acid (TFA)) expressed as PFOA equivalents [22] to be 4.4 ng/L (annual average). Despite this growing regulatory attention, PFAS monitoring still largely depends on targeted analysis, which is limited to compounds with available analytical reference standards [23,24]. Currently, fewer than 300 PFAS standards exist, while the number of structurally diverse PFAS in use exceeds 10,000. The recently developed EPA Draft Method 1633 [25] provides one of the most comprehensive targeted protocols, covering 40 PFAS in multiple environmental matrices by LC–MS/MS. The limited scope of these methods highlights the importance of SSA and NTA for detecting previously unrecognized PFAS.

In light of the forthcoming revision of EU surface water legislation, which introduces monitoring requirements for 25 PFAS [22], Serbia, currently aligning its environmental policies with EU directives, faces both regulatory and infrastructural challenges. Major cities such as Belgrade, Novi Sad, and Niš, representing nearly one-third of the national population, still lack functional wastewater treatment plants [26]. Since conventional wastewater treatment does not effectively remove PFAS, comprehensive investigations combining targeted and suspect screening are crucial for establishing a national baseline and guiding future policy actions.

The objectives of this study were, therefore, to (1) perform targeted analysis of 25 PFAS in Serbian surface waters characterized by varying hydrological conditions and anthropogenic pressures; (2) apply SSA using HRMS to identify and semi-quantify PFAS beyond the targeted list; (3) classify the identified PFAS according to confidence levels following the Schymanski and Charbonnet frameworks [16,17] and assess their spatial distribution; and (4) evaluate potential ecological risks and prioritize compounds of concern. This work represents the first comprehensive PFAS screening in Serbian surface waters, combining targeted and SSA approaches and providing essential baseline data to support regulatory harmonization and environmental protection efforts.

## 2. Materials and Methods

### 2.1. Chemicals and Solvents

Native PFAS solution mixture (PFAS-MHX) and a mixture of isotopically labeled standards (MPFAC-HIF-ES), obtained from Wellington Laboratories (Guelph, ON, Canada), were used for targeted and SSA approaches. The native PFAS mixture contained perfluoroalkyl carboxylic acids, PFCAs (11 compounds); perfluoroalkyl sulfonic acids, PFSAs (8); fluorotelomer sulfonic acids (3); and perfluorooctane sulfonamides (3) (Supporting Materials, Table S1). Oasis^®^ WAX cartridges (150 mg, Waters^®^, Milford, MA, USA) were used for solid-phase extraction (SPE). LC–MS grade methanol and ammonium acetate (NH_4_Ac) were purchased from JT Baker (Mallinckrodt Baker, Phillipsburg, NJ, USA), while LC–MS grade ammonium hydroxide (25%, NH_4_OH) was obtained from the same supplier. Ultra-pure water (resistivity of 18.2 MΩ·cm at 25 °C, total organic carbon < 5 µg/L, and microbial count < 0.1 CFU/mL) was produced using a Direct-Q system (Millipore, Milan, Italy).

### 2.2. Sampling

**Figure 1:**
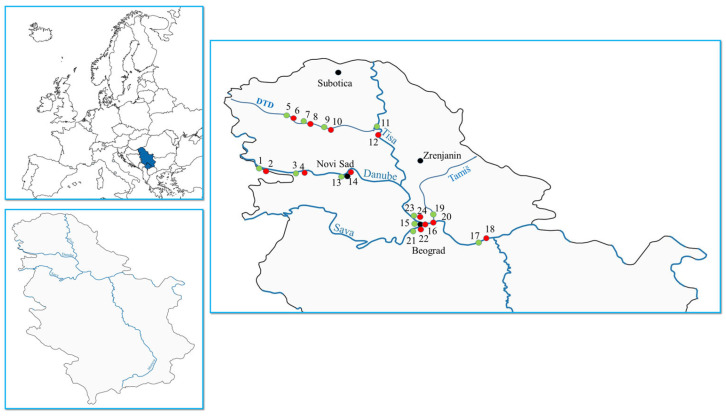
Geographical overview of the study area and sampling locations (QGIS 3.44.1). Even-numbered sampling sites correspond to locations downstream of settlement (i.e., after passing through a settlement), while odd-numbered sites are upstream before entering the settlement.

A comprehensive sampling campaign was conducted in November 2023 to assess the presence of PFAS in surface water bodies across northern Serbia. A total of 12 locations were selected within key aquatic ecosystems of the region; a geographical overview of the study area and sampling locations is presented in Figure 1, prepared by QGIS (Quantum Geographic Information System) software (version 3.44.1). The sampling covered 12 settlements along major rivers, including the village of Bačko Novo Selo (samples nos. 1 and 2, Figure 1), located on the Danube River where it enters Serbia from Croatia, selected as a reference area with relatively low anthropogenic pressure. To capture the potential influence of anthropogenic activities, surface water samples were collected both upstream and downstream of the settlements. The selected sites encompassed the main surface water bodies of the region: the Danube River (*n* = 6 settlements, Bačko Novo Selo, Bačka Palanka, Novi Sad, Zemun, Belgrade, Smederevo, *y* = 12 samples); the Great Bačka Canal, the main component of the Danube–Tisa–Danube (DTD) irrigation system (*n* = 3, *y* = 6, Kula, Vrbas, Srbobran); the Tisa River (*n* = 1, *y* = 2, Bečej); the Tamiš River (*n* = 1, *y* = 2, Pančevo); and the Sava River (*n* = 1, *y* = 2, Novi Beograd). A detailed overview of all sampling sites, including sampling coordinates and site characteristics, is available in the Supporting Materials (Text S1, Table S2). The selected sites represent diverse hydrological and land-use conditions characteristic of northern Serbia, including urban, industrial, and agricultural areas along major river systems. Sampling locations were strategically chosen to capture both background and anthropogenically influenced sections, particularly those affected by municipal and industrial wastewater discharges.

Surface water samples (2 L) were collected in pre-cleaned high-density polypropylene (HDPP) bottles, which were rinsed on-site with the sampled water before collection to minimize contamination. All samples were transported to the laboratory on the same day in cooled containers maintained at 4 °C. Within 24 h of collection, SPE was conducted. Before SPE, samples were filtered using a 45 mm MN 85/70 BF filter paper (Macherey-Nagel, Düren, Germany) to remove suspended solids and particulate matter.

### 2.3. Sample Preparation

The sample preparation protocol was based on a previously published method by Barbosa et al. [7]. In brief, SPE was performed using Oasis^®^ WAX cartridges. The cartridges were placed on a vacuum manifold (PerkinElmer, Waltham, MA, USA) and conditioned sequentially with 5 mL of methanol and 5 mL of ultrapure water. Following conditioning, 200 mL of previously filtered surface water was loaded onto each cartridge at a flow rate of approximately 1 mL/min. After sample loading, the sorbents were dried by passing ambient air through the cartridges for one hour. Subsequently, the cartridges were further dried under vacuum for an additional 45 min. PFAS were eluted in two steps (2 × 4 mL) with methanol containing 0.1% NH_4_OH, which was passed through the cartridges into collection vials. The eluates were evaporated under a gentle stream of nitrogen at 40 °C to near dryness. The residues were then transferred to LC vials equipped with 250 µL inserts, further dried, and reconstituted in 100 µL of ultrapure water/methanol (90:10, *v*/*v*). SPE blanks were included in each extraction batch to monitor and prevent cross-contamination.

### 2.4. Instrumental Analysis

The performance characteristics of the instrumental method used in this study were previously validated and described by Barbosa et al. [7]. Chromatographic separation was performed using an Acquity LC system (Waters, Milford, MA, USA) equipped with a Hypersil GOLD PFP LC C18 analytical column (50 × 3 µm; Thermo Fisher Scientific, San Jose, CA, USA). The column was maintained at 40 °C to enhance chromatographic efficiency and retention time reproducibility. The mobile phases consisted of (A) 20 mM aqueous ammonium acetate and (B) 20 mM ammonium acetate in methanol. The gradient elution program was as follows: the initial composition of 20% B was linearly increased to 80% B over 5 min, followed by a ramp to 90% B within the next 5 min. This composition was held for 2 min to ensure complete elution of late-eluting compounds. The gradient was then returned to the initial conditions (20% B) within 1 min and maintained for an additional 1 min for column re-equilibration. The total run time was 12 min per injection, with a constant flow rate of 0.200 mL/min. An injection volume of 10 µL was used to balance sensitivity and minimize matrix effects.

Mass spectrometric detection was carried out using a Q Exactive™ hybrid quadrupole-Orbitrap mass spectrometer (Thermo Fisher Scientific, San Jose, CA, USA), operated in negative electrospray ionization (ESI) mode. Data were acquired in full scan (FS) mode across an m/z range of 90–1500, with a resolving power of 70,000 FWHM (full width at half maximum, FWHM). Additionally, data-dependent MS^2^ (ddMS^2^, DDA) acquisition was performed on the most intense precursor ions, using a resolution of 15,000 FWHM, enabling structural elucidation and confident compound identification. The instrumental setup was configured to support both suspect screening of PFAS and targeted quantification of 25 previously optimized compounds. Instrument control, data acquisition, and processing (targeted analysis) were conducted using Xcalibur software (Thermo Fisher Scientific, San Jose, CA, US, version 4.6.67.17). System suitability tests, including checks for retention time stability, mass accuracy, and peak shape, were routinely performed to ensure consistent analytical performance. Calibration and quality control (QC) standards were injected regularly to verify data reliability and reproducibility throughout the analytical sequence. Mass-labeled standards (Table S1) were added to all samples at final concentrations ranging from 3.12 to 2.00 ng/L (depending on the PFAS concentration in the original internal standard mixture) prior to analysis. Method validation parameters are provided in the previous study [7].

### 2.5. Data Processing

Quantitative analysis of 25 PFAS was performed applying calibration curves generated from reference standards, resulting in Level 1 confidence identification according to Schymanski et al. [16]. Internal calibration curves were constructed using standard solutions across the concentration range of interest. For compounds lacking a specific isotopically labeled internal standard, quantification was carried out using the most structurally similar labeled compound. Similarity was assessed based on molecular weight, retention time, and functional groups to minimize quantification bias.

**Figure 2:**
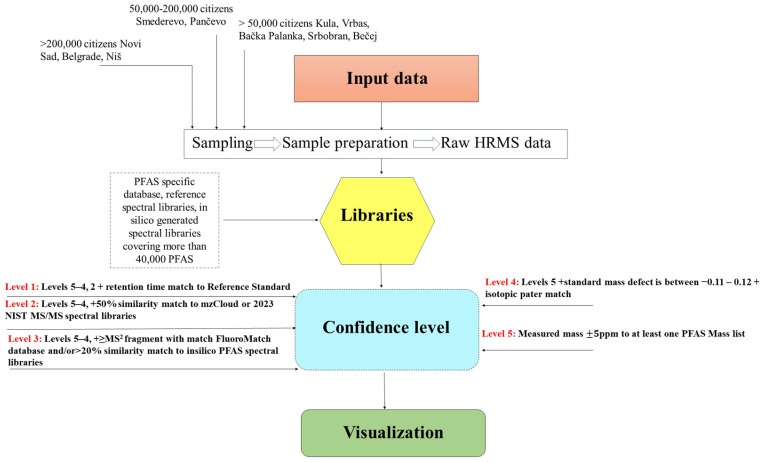
Workflow for suspect screening analysis of PFAS.

To identify PFAS not included in target analysis, SSA of Orbitrap raw data was conducted using Compound Discoverer™ (CD) software (3.5 beta version, Thermo Fisher Scientific, San Jose, CA, US). The data were processed through customized modular workflows tailored for PFAS analysis, enabling feature extraction and compound identification [2]. Detailed parameters of each processing node and workflow settings are provided in the Supplementary Material (Table S3), while Figure 2 presents a schematic overview of the main steps and key modules within the SSA workflow. SSA of surface water extracts was used to identify PFAS and assign annotation confidence levels (L1–L5) based on the framework proposed by Schymanski et al. [16]. Level 1 represents confirmed identification using an authentic reference standard (matching accurate mass, retention time, and MS/MS spectra), while Levels 2 and 3 correspond to probable and tentative structures supported by diagnostic fragments and library or in silico evidence. Level 2 annotations were assigned based on high-confidence matches to curated reference spectral libraries, including the Thermo Scientific™ mzCloud™ advanced mass spectral database and the 2023 NIST Tandem Mass Spectral Library. Levels 4 and 5 indicate decreasing certainty, ranging from unequivocal molecular formula assignment to features defined only by accurate mass. Level 3 annotations were assigned when PFAS initially classified at Level 4 exhibited MS/MS fragment information, with at least one measured fragment matching entries in the Duke University in silico PFAS spectral library or the FluoroMatch PFAS fragment database. The SSA workflow (Figure 2, Table S3) included the following analytical steps. First, raw LC–HRMS data were imported into CD and processed via a peak picking algorithm that includes chromatographic alignment (adaptive curve was used as alignment model with the maximum shift time of 2 min), grouping of features across files (RT tolerance of 0.2 min), assembling compounds from detected features (preferred MS^2^ ions [M-H]^−1^), and blank subtraction to reduce background noise (maximum allowed ratio of the sample vs. blank to be considered as background was set to 5). Unknown features were detected by peak deconvolution and grouped by retention time, exact mass (mass tolerance ±5 ppm), and isotopic pattern similarity (matching threshold ≥ 80%). Features displaying halogen-specific isotopic signatures were prioritized. Molecular formulas were assigned using a mass tolerance of ±5 ppm, isotope intensity tolerance of ±30%, and heuristic rules favoring elemental compositions consistent with typical PFAS structures (C, H, F, O, S, P, Br, and Cl). Detected features were screened against publicly available PFAS suspect lists (both the NIST Suspect List of PFAS and the EPA PFAS Structure List) and the curated PFAS suspect list. Matching criteria included precursor ion mass (±5 ppm), theoretical isotopic pattern, and, where available, retention time from authentic reference standards. For features with associated MS^2^ spectra, experimental fragmentation patterns were compared against the Thermo Scientific™ mzCloud™ and 2023 NIST MS/MS reference spectral libraries as well as the PFAS-specific in silico generated spectral library [27]. A database containing > 800 diagnostic PFAS fragments, such as CF_3_^−^, C_2_F_5_^−^, and SO_3_^−^, and an additional database containing PFAS-specific neutral losses were used to support structural identification. For each detected feature, the standard mass defect, Kendrick Mass Defect (KMD; using CF_2_ and CH_2_ Kendrick formulas), and additional values used for orthogonal plots (mass defect and molecular mass values normalized by carbon number) were automatically calculated. Both orthogonal plots (referred to as “PFAS Plot”) and KMD plots are generated within the CD software and help distinguish PFAS from non-fluorinated organic compounds (PFAS Plot) and identify multiple PFAS that belong within the same homologous group (KMD plot). Annotations were classified according to the system proposed by Schymanski et al. [16], assigning confidence levels from Level 1 (confirmed structure with match to retention time of an authentic reference standard) to Level 5 (match to an exact mass within at least one suspect list only). Only features with confidence levels 1–3 were retained for interpretation. All tentative identifications were manually reviewed to exclude false positives, background artifacts, and low-quality features. Final reports prioritized substances based on analytical confidence, detection frequency, and environmental relevance. This suspect screening strategy enabled semi-automated, confident identification of both known and emerging PFAS, providing a robust framework for comprehensive chemical characterization of surface water samples from complex environmental matrices.

Following identification through the SSA, the concentration of the majority of PFAS was estimated through a semi-quantitative approach developed previously [24]. Quantification of target PFAS was performed using the isotope-dilution method, where relative response factors (RRFs) were determined for each compound based on calibration with authentic native and isotopically labeled standards (Table S1). These experimentally derived RRFs were then applied to semi-quantify PFAS identified through the SSA workflow by assigning the most appropriate surrogate RRFs according to similarities in retention time and molecular structure between SSA-identified and target compounds [28,29].

During quantification, good laboratory practices were strictly followed to minimize potential background contamination. In cases where PFAS compounds were detected in procedural blanks, blank corrections were applied. Quantification was performed only for compounds showing an S/N greater than 3, an uncertainty in the qualifier-to-quantifier ion ratio below 30%, and a retention time deviation within ±0.1 min for the 25 PFAS quantified with authentic standards.

### 2.6. Risk Modeling and Prioritization

To prioritize the identified PFAS according to their potential environmental risk, a three-step risk assessment framework was applied: (1) hazard scoring based on persistence, bioaccumulation, and toxicity (PBT); (2) exposure evaluation combining detection frequency (DF) and concentration magnitude; and (3) integration of hazard and exposure into a composite risk index (RI) for ranking [23,24]. The PBT properties of each compound (18 attributes in total) were predicted using a suite of quantitative structure–activity relationship (QSAR) models, including EPI Suite v4.11, ECOSAR v2.0, T.E.S.T. v5.1.1, VEGA v1.2.0, and QSAR Toolbox v4.5, in accordance with REACH Annex XIII and GHS classification criteria (Table S4). Persistence (P) was modeled using the Biowin biodegradation suite (Biowin1, 3, and 5) within EPI Suite v4.11, which estimates the probability and timeframe of aerobic biodegradation and readiness under MITI test conditions. Bioaccumulation (B) was characterized by the bioconcentration factor (BCF) from BCFBAF v3.01 and the octanol–water partition coefficient (Log Kow) from KOWWIN v1.68, both log-transformed [log_10_(x)] for normalization. Toxicity (T) encompassed both ecotoxicological and human health endpoints. Acute aquatic toxicity (fish LC_50_ 96 h, Daphnia LC_50_ 48 h, and green algae EC_50_ 96 h) was modeled using ECOSAR v2.0 and expressed as –log_10_(mg/L) to standardize toxicity potency. Human health toxicity included carcinogenicity, developmental toxicity, mutagenicity, endocrine disruption, hepatotoxicity, skin/eye irritation or corrosion, sensitization, and repeated-dose toxicity, predicted using VEGA v1.2.0, QSAR Toolbox v4.5, and IRFMN models (CAESAR, COMPARA, VERMEER, CERAPP, CORAL, Antares, and ISS). Acute mammalian toxicity was represented by the oral rat LD_50_, modeled in T.E.S.T. v5.1.1. All attributes were normalized and weighted according to their relative importance (1/9 for persistence, 1/6 for bioaccumulation, 1/18 for ecotoxicological, and 1/60 for human toxicity endpoints) to ensure balanced integration across criteria (Figure S1). The combined weighted scores were aggregated to generate a Toxicological Priority Index (ToxPi) for each PFAS [30], providing a quantitative hazard ranking. Exposure assessment was then incorporated through DF (reflecting detection frequency) and concentration magnitude, normalized according to Equation (1). The final risk index (RI) integrated the normalized hazard and exposure components, enabling comparative prioritization of PFAS according to their overall environmental risk potential. Secondly, exposure was quantified by calculating DF and concentration magnitude, reflecting relative intensity. Concentration magnitude was normalized using Equation (1):

(1) $$\mathrm{Magnitude}=\frac{C_{i}\;-\;C_{\min}}{C_{\max}\;-{\;C}_{\min}}$$

where C*_i_* is the maximum concentration of PFAS*i*; C_max_ is the highest value among the maximum concentrations of all PFAS; and C_min_ is the lowest value among the maximum concentrations of all PFAS. Exposure was estimated as (Equation (2)):

Exposure = DF × Magnitude(2)

and then normalized to a 0–1 scale by Equation (3):

(3) $${\mathrm{Exposure}}_{\mathrm{normalized}}\;=\;\frac{{\mathrm{Exposure}}_{i}\;-\;{\mathrm{Exposure}}_{\min}}{{\mathrm{Exposure}}_{\max}\;-\;{\mathrm{Exposure}}_{\min}}$$

where Exposure*_i_*, Exposure_min_, and Exposure_max_ represent the exposure value for PFAS*i* and the minimum and maximum exposure values among all PFAS, respectively.

Thirdly, the final risk index (RI) was calculated by combining the normalized exposure with the ToxPi hazard score (Equation (4)):

RI = ToxPi Score × Exposure_normalized_ (4)

Using the RI values, PFAS compounds were classified into four categories of risk: high (RI > 0.1), medium (0.01 < RI ≤ 0.1), low (0.001 < RI ≤ 0.01), and negligible (RI ≤ 0.001). This comprehensive risk assessment framework facilitates the efficient identification and ranking of PFAS substances that pose the highest threats to environmental and public health management.

### 2.7. Use of AI Tools

During the preparation of this manuscript, the authors used ChatGPT (OpenAI, 5.1, 2025) for the purpose of improving the clarity of the English language. The authors have reviewed and edited the output and take full responsibility for the content of this publication.

## 3. Results and Discussion

### 3.1. Targeted Analysis

**Figure 3:**
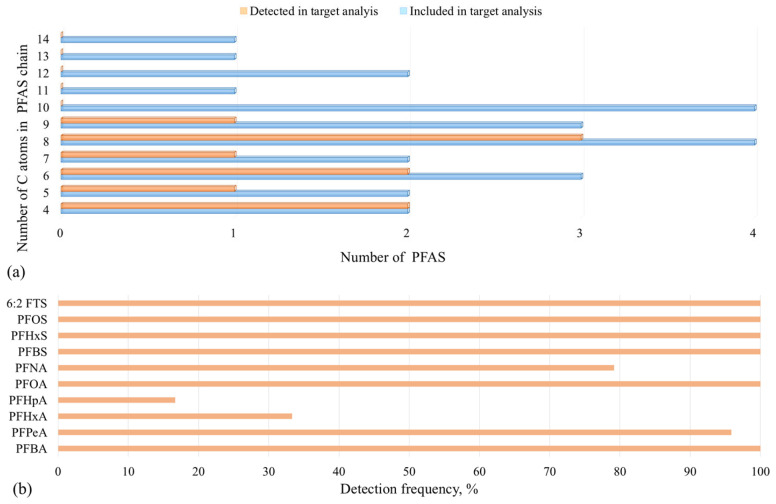
Occurrence of PFAS in surface water samples: (**a**) number of PFAS included in the target list and quantified in the samples, grouped by perfluoroalkyl carbon-chain length; (**b**) detection frequency (%) of 10 individual PFAS quantified in this study (out of 25 targeted analytes).

Among the 25 targeted PFAS compounds analyzed in this study, a total of 10 compounds were quantified across the surface water samples collected from Serbian rivers (Figure 3), as summarized in Tables S5 and S6. Figure 3a shows that, although the targeted list covered PFAS across a wide range of chain lengths (C4–C14), the targeted analysis quantified PFAS only in the C4–C9 range, with no detections of the targeted C10–C14 PFAS above method quantification limits (MQLs) in the analyzed samples. The most frequently quantified PFAS had generally DFs above 75%, with the exception of PFHpA and PFHxA, which showed DFs of 16.7% and 33.3%, respectively (Figure 3b, Table S6). DFs further indicated that several emerging short-chain PFAS (3 < C < 8) were widespread (e.g., PFBA, PFBS, and PFHxS had a DF of 100%, while the DF of PFPeA was 95.8%), consistent with their high water solubility and mobility in surface waters. Notably, PFOA, PFOS, and 6:2FTS were detected in all samples (DF 100%), indicating persistence and/or continuous inputs. The non-detection of the targeted C10–C14 PFAS indicates that, if present, their dissolved-phase concentrations were below MQL in the analyzed samples; if present, these compounds may also preferentially partition to suspended particulates and sediments rather than remain in the dissolved water phase. This distribution highlights this study’s balanced inclusion of both legacy and emerging PFAS, enabling an assessment of environmental occurrence trends across different chain lengths.

The sum of quantified PFAS (ΣPFAS_quant_) across the investigated surface water systems ranged from 2.92 to 52.6 ng/L (Table S6). Although ΣPFAS_quant_ may underestimate the total PFAS burden, 15 of the 25 targeted PFAS were not quantified in any of the samples. Accordingly, under a lower-bound approach (non-quantified targets set to 0), ΣPFAS_25_ equals ΣPFAS_quant_ for each sample. This consistent non-detection is an important finding, indicating that 15 compounds were not present at quantifiable levels under the studied conditions, particularly given that the limits of quantification applied in this study are comparable to those reported in the literature for similar matrices and analytical approaches [23,24].

To enable comparison with the proposed surface water EQS of 4.4 ng/L (expressed as PFOA equivalent) [22], the PFOA equivalent concentration for each quantified PFAS was calculated by multiplying its measured concentration by a compound-specific relative potency factor (RPF, Table S6), which accounts for differences in toxicological potency relative to PFOA. The resulting PFOA equivalents for all PFAS quantified at each location were then summed to obtain ΣPFOA_eq(quant)_ for each site, which was compared directly with the proposed PFOA_eq_-based EQS [22]. ΣPFOA_eq(quant)_ ranged from 1.6 to 19.4 ng/L (mean 6.7 ng/L, median 6.2 ng/L) across the 24 sampling sites (Table S6) and exceeded the proposed group EQS of 4.4 ng/L at 17/24 sites. The contribution of measured PFOA content to the overall ΣPFOA_eq(quant)_ was about 20% (mean and median 0.2; maximum 0.7, Table S6), indicating that exceedances above 4.4 ng/L were largely driven by other PFAS captured by the equivalency approach rather than by PFOA alone. Only at one location, individual PFOA concentration (6.1 ng/L, Figure S2) exceeded the proposed PFOA EQS. The PFOA_eq(quant)_ burden across the 24 samples was mainly dominated by PFNA and PFOS, with their median contributions of about 45% and 31%, respectively. Figure S2 corroborates that the site-specific PFOA_eq_ profiles are dominated by PFNA and PFOS (and occasionally by PFOA) and shows that, at several locations, the individual PFOA_eq_ values of these compounds alone already exceeded the proposed group EQS threshold of 4.4 ng/L.

**Figure 4:**
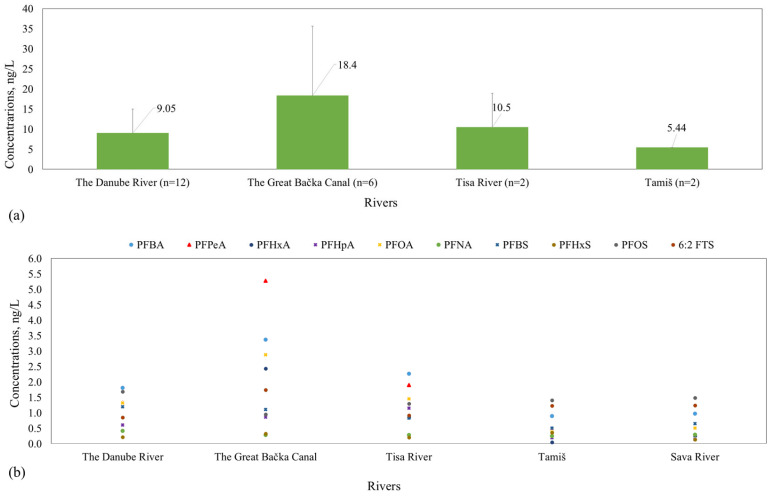
The distribution of PFAS: (**a**) the mean and standard deviation of total PFAS concentration (ΣPFAS_quant_) per river and (**b**) the average concentrations of individual PFAS in Serbian rivers.

The Great Bačka Canal (GBC) exhibited the highest average total concentration (Figure 4a), 18.4 ng/L (six locations), as well as the highest maximum value (52.6 ng/L), indicating contamination likely due to intensive anthropogenic (urban and/or industrial) pressures (Table S2) as well as poor dilution capacity typical for canal systems. Several food-processing industries, including the confectionery industry, meat and meat-processing industry, sugar production, and edible oil manufacturing, are located along the Great Bačka Canal between the Kula and Vrbas settlements. The Danube River water showed a range of 2.92–22.2 ng/L (12 locations) for ΣPFAS_quant_ concentration, with an average of 9.05 ng/L, which is lower than that found for GBC. The Tisa River water exhibited a similar ΣPFAS_quant_ average concentration as the Danube (10.5 ng/L, two locations), in a concentration range of 4.53–16.5 ng/L. The Sava and Tamiš rivers exhibited the lowest average ΣPFAS_quant_ concentrations, which were both about 6 ng/L (i.e., 5.31 ng/L (two locations) and 5.44 ng/L (two locations), respectively). In the Tamiš River, almost identical ΣPFAS_quant_ concentrations were observed upstream and downstream of Pančevo (5.42ng/L and 5.45 ng/L, respectively), suggesting that any local sources (if present) did not produce a discernible concentration gradient between the two points during the sampling event (~30 min apart). Overall, the observed concentration profiles indicate that semi-closed systems such as GBC are strongly driven by nearby emission sources, resulting in pronounced small-scale variability and sharper concentration gradients, whereas high-flow river sections have greater dilution and dispersion capacity, leading to attenuated concentrations. These findings highlight the need for differentiated monitoring and mitigation strategies across various water bodies, considering their hydrological and anthropogenic contexts.

Figure 4b presents an overview of the average concentrations of individual PFAS detected in surface water across all sites/samples analyzed. In 17 samples, PFOS concentrations surpassed the EU AA-EQS for inland surface waters of 0.65 ng/L, while the maximum allowable concentration (MAC-EQS: 36 µg/L) was not exceeded in any sample [21]. As observed, the average concentrations of individual PFAS were generally equal or less than 2 ng/L, except in the case of GBC, where the average levels were up to almost 5.5 ng/L (5.27 ng/L PFPeA, 3.37 ng/L PFBA, and 2.87 ng/L PFOA). Again, it could be said that the differences in levels suggest an influence of local point sources, possibly linked to industrial discharges, wastewater effluents, and agricultural runoff [7]. The presence of high PFPeA and PFBA concentrations, both short-chain PFCAs, supports their classification as “emerging” contaminants due to their increased usage as substitutes for regulated long-chain analogues. The highest average levels of individual PFAS in the Danube River were found for PFBA (1.81 ng/L), PFPeA (1.32 ng/L), and 6:2 FTS (1.68 ng/L). In the Tisa River, PFBA (2.25 ng/L) and PFPeA (1.89 ng/L) dominated the PFAS profile, while long-chain PFOA and PFOS were also consistently detected.

In Tamiš, the highest average was found for 6:2 FTS (1.40 ng/L), followed by PFOS (1.22 ng/L), PFBA (0.884 ng/L), and PFOA (0.380 ng/L). Interestingly, the Sava River water, while generally showing lower concentrations of short-chain PFCAs (e.g., PFPeA: 0.204 ng/L; PFHxA: 0.246 ng/L), had levels of PFOS (1.23 ng/L) and 6:2 FTS (1.48 ng/L) comparable to the Tamiš, aligning with findings in other moderately impacted European rivers [7].

Short-chain PFAS are characterized by water solubility and mobility, which facilitate their widespread dispersion in surface water systems. In contrast, long-chain PFCAs and PFSAs (e.g., PFNA, PFOS) were present at lower concentrations but still frequently detected, illustrating their persistence, bioaccumulation potential, and ongoing environmental concern despite regulatory controls. The widespread detection of 6:2 FTS, with rather similar average concentrations across samples (ranging from 0.943 ng/L in GBC to 1.68 ng/L in the Danube River), is noteworthy. As a replacement compound introduced to reduce the environmental burden of PFOS-based products [31], its frequent detection raises concerns about the environmental behavior and toxicity of such substitutes, which may not be fully understood or regulated.

**Figure 5:**
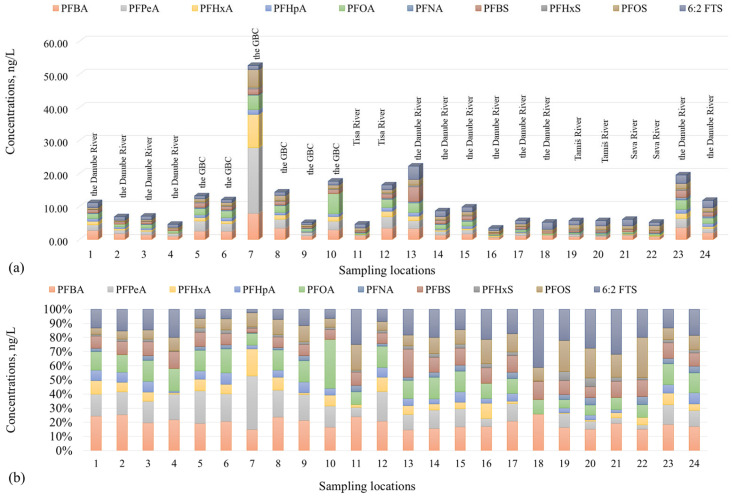
Distributions of PFAS across the sampling locations (1–24, described in Table S2): (**a**) concentrations of individual PFAS at investigated locations and (**b**) relative composition (% of ΣPFAS_quant_) at each site.

Distributions of individual PFAS across each sampling location are presented in Figure 5. At 14 locations, ΣPFAS_quant_ was below 10 ng/L; at nine, it was between 10 and 23 ng/L; and, at only at one location, it was up to around 50 ng/L, which indicated differences in local pollution sources, hydrological conditions, and potential dilution effects. The highest ΣPFAS_quant_ load was recorded for sample no. 7 near Vrbas (52.6 ng/L), driven by very high concentrations of PFPeA (19.9 ng/L) and PFHxA (10.0 ng/L), indicating a significant point source likely linked to industrial activity or untreated wastewater (Table S2). A considerable decrease was then observed at site 8 (Vrbas downstream, 13.6 ng/L). Similarly, at Novi Sad, a relatively elevated level at site 13 (upstream, 22.2 ng/L) was followed by a marked reduction downstream at site 14 (7.94 ng/L), likely reflecting dilution and/or changes in pollution inputs. An opposite trend was observed at Srbobran, where ΣPFAS_quant_ was higher downstream (sample no. 10: 16.8 ng/L) than upstream (no. 9: 4.64 ng/L), suggesting additional inputs along the reach. According to the site characterization provided in the Supplementary Material (Table S2), the downstream section is influenced by agricultural activities and the presence of a small packaging industry, which may represent potential but unconfirmed sources of PFAS. At Bečej, ΣPFAS_quant_ likewise increased from upstream (4.53 ng/L) to downstream (16.5 ng/L), with PFBA and PFPeA as dominant compounds. At Kula, upstream sample no. 5 (12.4 ng/L) and downstream sample no. 6 (12.0 ng/L) showed similar PFAS concentrations, indicating relatively consistent inputs along this section of the watercourse. Near Bačko Novo Selo, a small settlement without significant industrial activity, concentrations at both sampling points (10.3 ng/L and 5.90 ng/L) were comparable to those measured near larger settlements, suggesting inputs from upstream and/or non-industrial local sources, such as domestic wastewater and agricultural runoff (Table S2). This highlights the complexity of PFAS occurrence patterns even where direct anthropogenic pressures are limited. Samples no. 3 and 4 near Bačka Palanka also showed a modest decline from upstream (6.04 ng/L) to downstream (4.46 ng/L).

Urban centers such as Belgrade, Pančevo, and Smederevo generally exhibited lower to moderate ΣPFAS_quant_ (≈5–10 ng/L) in this study, with minimal upstream–downstream differences; the lowest total concentration was 2.92 ng/L at Belgrade (downstream). Given the absence of wastewater treatment plants, observed downstream reductions are most plausibly due to dilution rather than treatment and may also reflect spatial variability in source locations.

Considering that samples were collected upstream and downstream of each settlement, statistical analyses were conducted to assess the presence of statistically significant differences in PFAS concentrations between pre- and post-settlement locations (i.e., systematic differences in PFAS concentrations between paired pre- and post-settlement locations were tested). For each PFAS, upstream and downstream concentrations were compared across settlements using the paired non-parametric Wilcoxon signed-rank test, consistent with the matched (paired) sampling design. Normality of paired differences was evaluated using the Shapiro–Wilk test. To account for multiple comparisons across the set of PFAS, *p*-values were adjusted using a false discovery rate (FDR) procedure. After FDR correction, no statistically significant upstream–downstream differences were observed for any investigated PFAS.

### 3.2. Comparative Analysis of Maximum PFAS Concentrations Across European Studies

The comparative overview of maximum concentrations of targeted PFAS in European surface waters (Table S7) reveals pronounced spatial differences among freshwater systems across Europe, reflecting variability in industrial activity, regulatory enforcement, and wastewater treatment performance [7,32,33,34,35,36,37]. While cross-study comparisons should be interpreted cautiously due to differences in sampling design, analytical scope, and reporting conventions, this overview enables clearer benchmarking of upper-end PFAS concentrations among European surface waters. Table S7 reports the number of targeted analytes (*N*) for each study and expresses the reported maximum total content as ΣPFAS*_N_*, thus allowing a conservative, worst-case comparison. Maximum ΣPFAS*_N_* values range from 35.02 ng/L in Malta (*N* = 7) [37] and 42 ng/L in Finland (*N* = 23) [36] to substantially higher maxima in France (ΣPFAS*_N_* = 725 ng/L; *N* = 22) [33] and Spain (ΣPFAS*_N_* = 2878 ng/L; *N* = 21) [32].

In several highly impacted systems, long-chain PFAS, particularly PFOS and PFOA, contribute substantially to the overall PFAS burden. Exceptionally high PFOS concentrations were reported in Spain (2709 ng/L) [32] and France (197 ng/L of branched-PFOS and 173 ng/L of linear-PFOS) [33], with the latter accompanied by elevated PFHxS levels (217 ng/L) [33], indicating strong localized contamination likely linked to historical industrial emissions and legacy uses of PFOS-containing products, including firefighting foams [32,33,34,35]. In the study by Llorca et al., [32] an exceptionally high PFOS concentration (2709 ng/L) was reported at a single Spanish surface water site. While no site-specific source attribution was provided, the authors associated such elevated levels with highly industrialized areas, suggesting industrial activities as the most plausible contributors rather than diffuse urban pollution. Elevated concentrations of long-chain PFCAs, such as PFDA (213 ng/L) and PFNA (52 ng/L), further underline the influence of persistent legacy sources in Spain surface waters [32].

In contrast, northern Serbia exhibits a distinct PFAS profile dominated by short-chain perfluoroalkyl carboxylic acids, with PFPeA (19.9 ng/L), PFHxA (10.0 ng/L), and PFBA (7.86 ng/L) representing the major contributors, while PFOS (5.3 ng/L) and PFHxS (0.57 ng/L) occur at comparatively low concentrations. A similar predominance of short-chain PFAS is observed in several other European regions, including Spain (PFBA up to 125 ng/L), Portugal (PFBA 23 ng/L), and Germany (PFBA 23 ng/L), supporting the notion of a broader European transition toward short-chain PFAS as substitutes for regulated long-chain compounds.

Overall, the data summarized in Table S7 place northern Serbia among the less contaminated European freshwater systems, characterized by moderate maximum total levels of quantified PFAS and profiles dominated by short-chain PFCAs rather than legacy sulfonates. Such patterns are consistent with diffuse contamination sources, including municipal wastewater effluents and urban runoff, rather than strong point-source industrial inputs and align with recent observations reported for other moderately impacted European river systems.

### 3.3. Untargeted PFAS Workflow in Compound Discoverer 3.5

Application of the untargeted PFAS workflow in CD 3.5 initially yielded many molecular features not belonging to the PFAS class. Although outside this study’s primary scope, these non-PFAS features provide valuable insight into the broader chemical (contamination) profile at the investigated sites. This underlines one of the major strengths of non-targeted and suspect screening approaches, which is the capability to retrospectively mine HRMS datasets for additional compound classes or substances not considered during the initial analysis.

Following processing with the CD 3.5 workflow parameters (Table S3), data were refined using the Data Filter tool to retain only PFAS-related features. A detailed evaluation of the filtered compounds was then performed to ensure reliable identification, following the confidence-level framework of Schymanski et al. [16]. In total, 20 PFAS were identified through the SSA workflow (Tables S6 and S8), of which 10 were not part of the targeted quantification list of 25 PFAS. Five compounds were assigned at confidence level 2, and five were assigned at level 3 (Figure 2; Table 1, Tables S6 and S8). SSA-identified PFAS comprised two PFCAs (trifluoroacetic acid, TFA, and perfluoropropanoic acid, PFPrA) and one PFSA (trifluoromethanesulfonic acid, TFMS), representing ultra-short chain PFAS. Two hydrogen-substituted perfluorocarboxylic acids (7H-perfluoroheptanoic acid (7H-PFHpA) and 9H-hexadecafluorononanoic acid (9H-PFNA)) and one perfluoroether carboxylic acid (ADONA) were also detected. Additionally, flupropanate (2,2,3,3-tetrafluoropropionic acid), a hydrogen-substituted polyfluorocarboxylic acid used as a fluorinated pesticide, was identified. Flupropanate is not approved at the EU level under Regulation (EC) No 1107/2009 and is not registered for use in Serbia; however, limited emergency authorizations (Article 53) have been reported in certain Member States, notably Hungary and Croatia, in the form of its sodium salt (flupropanate-sodium). Other detected fluorinated pesticides (fluxapyroxad, isoxaflutole, fluometuron) are regulated at both the EU and national levels.

The PFAS identified through the SSA approach were further semi-quantified using the methodology proposed by Zhang et al. [24]. This semi-quantitative approach inherently involves a higher degree of measurement uncertainty since authentic analytical standards are not available for all compounds. Instead, RRFs from structurally and physicochemically similar PFAS, those exhibiting comparable retention times and ionization behavior, were applied (Table S8). Despite this limitation, the procedure represents a practical and cost-efficient strategy for estimating preliminary concentration ranges of PFAS not included in routine monitoring programs. Such information is valuable for screening purposes and supports prioritization of compounds that warrant further investigation and quantitative confirmation once authentic standards become available. In this context, semi-quantitative results should be interpreted as indicative values that provide an initial estimate of environmental occurrence rather than definitive concentrations. Nonetheless, when combined with confidence-level annotation and structural classification, these data deliver a comprehensive overview of PFAS contamination patterns across the studied sites and enable comparison between targeted and suspect screening outcomes.

Table 1 summarizes the PFAS compounds identified through the SSA workflow together with their structural information, annotation confidence levels, and semi-quantitative concentration ranges. For PFAS identified and semi-quantified through SSA, method quantification limits were estimated based on the lowest PFAS concentration in river waters, producing a signal-to-noise (S/N) ratio of 10 (Table S8) [38]. Among the ultra-short chain PFCAs, TFA was the most abundant, with concentrations ranging from 337 to 1165 ng/L (average 513 ng/L). The elevated TFA levels observed may originate from multiple sources. Given that there is no PFAS manufacturing in Serbia, plausible contributors include emissions from industrial and pharmaceutical uses, agricultural application of C–CF_3_-containing plant protection products, and secondary atmospheric formation through degradation of volatile fluorinated precursors and refrigerant gases [39,40,41]. Affricano et al. [41] reported average TFA concentrations of 0.48 µg/L in surface and well waters from Italy, comparable to the mean concentration obtained in this study (0.513 µg/L). In the same study by Affricano et al. [41], other ultra-short-chain PFAS, including PFPrA and TFMS, were analyzed but not detected. In this study, PFPrA and TFMS were detected at notable levels, with maximum concentrations of 51.3 ng/L and 11.2 ng/L, respectively. Although TFA was not considered in the ΣPFOA_eq(quant)_ values derived from targeted analysis, its high abundance and semi-quantified concentration range prompted an additional toxicity-weighted evaluation within the suspect screening framework. Using the RPF approach applied consistently with the target analysis (Table S6), TFA concentrations were converted to PFOA_eq_ and incorporated into an extended PFOA_eq_ assessment (ΣPFOA_eq(quant+SSA)_). When TFA-derived PFOA_eq_ were included, ΣPFOA_eq(quant+SSA)_ values ranged from 2.3 to 20.2 ng/L, with a mean of 7.6 ng/L and a median of 6.8 ng/L. Overall, 19 out of 24 sampling sites exceeded the proposed EU group EQS of 4.4 ng/L expressed as PFOA equivalents. These values are comparable to but slightly higher than those obtained from the targeted dataset alone, indicating that TFA contributes measurably to the overall toxicity-weighted PFAS burden (Table S6).

The detection of the ether-PFAS ADONA (4,8-dioxa-3H-perfluorononanoic acid) (DF = 75%) further indicates the presence of next-generation PFAS introduced as substitutes for legacy PFOA [42]. Although its average concentration was moderate (11.2 ng/L), its occurrence demonstrates the environmental dissemination of replacement perfluoroether acids. Research on ADONA remains limited; however, available studies indicate that its occurrence is most frequently reported in Germany, while concentrations in other regions are generally at trace levels. Pan et al. [38] quantified ADONA in surface water samples from major Chinese river systems. Reported concentrations ranged from <0.01 to 1.5 ng/L, with a low detection frequency (9%). In contrast, Joerss et al. [43] observed considerably higher levels, reporting an average ADONA concentration of 211.69 ng/L across four German rivers, suggesting possible localized industrial inputs or point-source discharges.

Hydrogen-substituted PFAS, namely 9H-PFNA (DF = 33.3%) and 7H-PFHpA (DF = 100%), were semi-quantified at low levels (<10 ng/L). Previous research has reported the occurrence of H–PFCAs in surface waters from the Netherlands [44]. Among these, 7H-PFHpA was quantified in one out of eleven surface water samples at a concentration of 0.3 ng/L, whereas 9H-PFNA was not detected in any of the analyzed samples. The most frequently detected compound was 4H-PFBA, with concentrations ranging between 1.4 and 2.0 ng/L [44]. The detection of ether-PFAS, such as ADONA, as well as hydrogen-substituted perfluoroalkyl carboxylic acids (H–PFCAs), namely 9H-PFNA and 7H-PFHpA, is not unexpected, as these compounds have been introduced as replacements for long-chain PFAS that are now regulated under environmental legislation. Although these substitutes were developed with the intention of being less bioaccumulative and toxic, recent studies have indicated that they may still exhibit considerable environmental persistence and potentially cause equal or even stronger adverse effects than legacy PFAS [44]. In contrast to long-chain PFAS, data on the occurrence of short-chain and ultra-short-chain analogues, such as those detected in this study, remain scarce, which makes these findings particularly valuable for understanding emerging PFAS contamination patterns.

Among the fluorinated pesticides, flupropanate was the only compound quantified (0.20–22.1 ng/L), whereas fluxapyroxad, isoxaflutole, and fluometuron were detected but not quantified. Due to their distinct chemical structures (Table 1), these compounds could not be matched with any native PFAS analytical standards used in the targeted method. All three possess aromatic structures, some containing multiple aromatic rings, where CF_3_ or HCF_2_ groups are attached to aromatic or other functional moieties. This structural configuration contrasts sharply with the predominantly linear perfluoroalkyl chains of target PFAS, in which fluorine atoms dominate the molecular backbone. As a result, these pesticides exhibit distinct chromatographic and ionization behaviors, preventing reliable quantification using PFAS-specific calibration standards. According to a global market analysis by Ogawa et al. [45], approximately 16% of all pesticides currently available on the market are fluorinated, highlighting the widespread use of organofluorine structures in modern agrochemicals. Even more concerning is the recent trend showing that nearly 70% of newly approved pesticides contain at least one fluorine atom in their molecular structure, underscoring the growing dependence of the agrochemical industry on fluorinated compounds [46,47]. Their degradation or transformation under biogenic or abiogenic conditions may contribute to the increasing background levels of TFA observed in environmental matrices. Fluxapyroxad and isoxaflutole are among the most extensively used and persistent fluorinated pesticides worldwide [46]. Fluometuron, also fluorinated, has been detected at elevated concentrations in monitoring programs globally, with values up to 0.176 mg/kg reported in vineyard soils in Spain [48].

Regarding the distribution of semi-quantified PFAS, Figure S3 illustrates the average concentrations (ng/L) detected across the five investigated rivers in northern Serbia. TFA dominated the PFAS profile, exhibiting concentrations one to two orders of magnitude higher than those of other compounds. Elevated levels of PFPrA, TFMS, and ADONA were observed in the Great Bačka Canal and the Tisa River, suggesting strong anthropogenic influence at these sites. In contrast, hydrogen-substituted PFAS (9H-PFNA and 7H-PFHpA) and the fluorinated pesticide flupropanate occurred at low concentrations (<20 ng/L), with the highest values again recorded in the Great Bačka Canal.

Although a significantly larger study [12,13], which included PFAS analysis across several countries (Germany, Austria, Czech Republic, Slovakia, Hungary, Slovenia, Croatia, Serbia, Romania, Bulgaria, and Ukraine), identified only 10 out of 56 targeted PFAS compounds through targeted analysis, an additional 72 compounds were detected using SSA, bringing the total number of PFAS identified to 82. 6:2 FTS was detected at concentrations ranging from 151 to 5101 ng/L, with a median value of 941 ng/L, while PFHxA was found at concentrations ranging from 1 ng/L to 11,894 ng/L, with a median value of 2296 ng/L; both compounds were detected far above the respective average concentrations found in the present study.

### 3.4. Risk Modelling and Prioritization of PFAS Compounds

**Figure 6:**
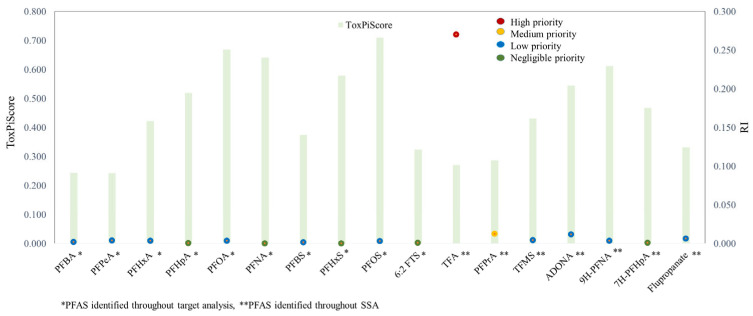
Calculated ToxPi and risk index (RI) values.

The quantified and semi-quantified PFAS were ranked according to 18 attributes reflecting their PBT properties in order to identify those posing the greatest potential threat to the environment and human health. This prioritization was conducted using the risk-based prioritization model developed and implemented by Hu et al. [23], which integrates both ecological hazards and human health risks into a single comprehensive assessment framework. Figure 6 and Table S9 summarize the results obtained during the step-by-step prioritization. Based on the obtained estimates (Table S9), three (PFOA, PFNA, PFHpA) out of the 20 quantified and semi-quantified PFAS can be categorized as PBT substances.

Based on estimated ToxPi scores, PFOS, PFOA, PFNA, and 9H-PFNA exhibited the highest hazard. The estimated ToxPi score (0.2428–0.7101) combined with normalized exposure (Equation (4)) ranked the PFAS according to their overall RI: one PFAS (TFA) with RI > 0.1 was identified as a high-priority substance, followed by PFPrA, which was classified as medium priority. Although PFOS, PFOA, and PFNA exhibited relatively high ToxPi scores (>0.600), reflecting their intrinsic toxicological relevance, they were classified as low-priority compounds based on the calculated RI. This outcome arises from the RI framework, which integrates not only toxicological weighting (ToxPi) but also exposure-related parameters, namely DF and concentration magnitude. While these compounds were detected with high DF across sampling sites, their concentrations were consistently low, resulting in limited overall risk scores. In contrast, TFA, despite having one of the lowest assigned ToxPi values, was classified as a high-priority substance due to its occurrence at concentrations one to two orders of magnitude higher than those of other quantified PFAS and high DF (100%), thereby substantially increasing its RI. In this way, PFAS compounds were prioritized for the main watercourses in Serbia, including an important canal network used for the irrigation of agricultural land.

The short-chain PFAS PFPrA is ranked among the highest-priority compounds, which underscores its environmental persistence, high bioaccumulation potential, and well-documented adverse effects. These findings are consistent with ongoing European regulatory actions, including the proposed universal PFAS restriction under the REACH framework, and reinforce the need for targeted monitoring and mitigation strategies in line with current policy directions [49].

From a regulatory perspective, the calculated risk RI should be interpreted as screening-level indicators rather than compliance metrics. RI values integrate measured or semi-quantified concentrations with available toxicity benchmarks, enabling the prioritization of PFAS for further monitoring, targeted assessment, or management action. Notably, TFA, identified here as the highest-priority compound, is included in the proposed revision of the EU surface water Water Framework Directive (WFD) that is currently in its final procedural stage [22]. Under this proposal, TFA is expected to be regulated within a group-based approach covering 25 PFAS, with concentrations expressed as PFOA-equivalents for screening-level compliance assessment. In contrast, PFPrA, classified as a medium-risk PFAS in this study, is not included in the current WFD proposal and is not part of the EU Watch List for potential prioritization [50]. This highlights the added value of risk-based prioritization approaches in identifying compounds of emerging concern that fall outside existing regulatory frameworks. Such prioritization is particularly relevant for non-EU countries, including Serbia, where EU environmental regulations are not implemented immediately and typically require transitional periods before adoption. In this context, RI-based evidence provides a scientifically grounded basis to support regulatory alignment, inform negotiations, and facilitate the more rapid and effective transposition of EU PFAS policies in candidate countries. Consequently, these results are valuable not only for raising societal awareness of PFAS contamination but also for supporting evidence-based decision-making by policymakers in Serbia.

## 4. Conclusions

In direct relation to this study’s objectives, targeted LC–HRMS successfully quantified 10 out of the 25 PFAS included in the target list in Serbian surface waters spanning contrasting hydrological conditions and anthropogenic pressures. Combining targeted LC–HRMS with SSA substantially broadened coverage beyond routine analyte lists: every targeted PFAS detected by quantification was also captured by SSA, and ~50% more PFAS were revealed overall, including ultra- and short-chain species and replacements. Confidence-level classification following the Schymanski and Charbonnet frameworks enabled the selection of PFAS suitable for reliable semi-quantification and subsequent risk prioritization. Semi-quantification and risk ranking identified TFA as the highest-priority compound, with one PFAS classified as medium priority, underscoring the need to look beyond legacy PFOS/PFOA toward newer chemistries. Spatial analysis indicated that semi-closed water bodies are especially sensitive to nearby inputs and show sharp within-reach variability, while high-flow rivers display more attenuated profiles, implying different monitoring and mitigation levers by water-body type. Although measured concentrations generally fall within the lower range reported for European rivers, frequent exceedances of the proposed 4.4 ng/L group threshold (expressed as PFOA equivalents) emphasize the regulatory relevance of toxicity-weighted contents for screening-level compliance. Overall, the first integrated targeted–suspect screening in Serbian surface waters provides decision-ready baseline data to support regulatory harmonization and environmental protection efforts and offers a practical template for harmonized surveillance across the Danube Basin as PFAS monitoring expands.

## Figures and Tables

**Table 1:** Summary of PFAS compounds identified by SSA, i.e, structural information, subclass, and semi-quantitative concentrations.

Annotation Name ^a^	Annotation Formula	Cas No	Class	NIST Suspect Id ^b^	CL ^c^	Structural Formula	Min, ng/L	Max, ng/L	Average, ng/L
TFA	C_2_HF_3_O_2_	76-05-1	PFCAs	3488	3	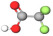	337	1165	513
PFPrA	C_3_HF_5_O_2_	422-64-0	PFCAs	3489	3	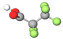	2.60	51.3	17.9
TFMS	CHF_3_O_3_S	1493-13-6	PFSAs	3490	3	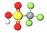	1.33	11.2	3.83
ADONA	C_7_H_2_F_12_O_4_	958445-44-8	perfluoroether carboxylic acid	2657	2	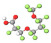	1.62	24.9	11.2
9H-PFNA	C_9_H_2_F_16_O_2_	76-21-1	hydrogen-substituted perfluorocarboxylic acid	3937	3	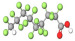	0.26	7.27	1.11
7H-PFHpA	C_7_H_2_F_12_O_2_	1546-95-8	hydrogen-substituted perfluorocarboxylic acid	2642	3	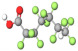	0.21	2.77	0.63
Flupropanate	C_3_H_2_F_4_O_2_	756-09-2	fluorinated pesticides	3302	2	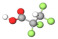	0.20	22.1	5.95
Fluxapyroxad	C_18_H_1_F_5_NO	907204-31-3	fluorinated pesticides	-	2	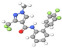	n.q	n.q	n.q
Isoxaflutole	C_15_H_12_F_3_NO_4_S	141112-29-0	fluorinated pesticides	-	2	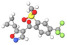	n.q	n.q	n.q
Fluometuron	C_10_H_11_F_3_N_2_O	2164-17-2	fluorinated pesticides	-	2	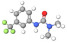	n.q	n.q	n.q

^a^ In most cases, multiple annotation sources had a direct match. The final annotations shown here were chosen to maintain naming consistency within a homologous class of PFAS. ^b^ NIST Suspect IDs were provided within the NIST Suspect List of PFAS Mass List used within the Compound Discoverer processing workflow. ^c^ Annotation confidence level assigned according to the Schymanski et al. [16] identification confidence criteria. n.q.—detected but not quantified, Trifluoroacetic acid (TFA), Perfluoropropionic acid (PFPrA), Trifluoromethanesulfonic acid (TFMS), 4,8-Dioxa-3H-perfluorononanoic acid (ADONA), 9H-Hexadecafluorononanoic acid (9H-PFNA), 7H-Perfluoroheptanoic acid (7H-PFHpA).

## Data Availability

Data are contained within the article and Supplementary Materials.

## Supplementary Materials

The following supporting information can be downloaded at: https://www.mdpi.com/article/10.3390/toxics14010078/s1, Text S1: Detailed description of sample locations; Figure S1: Risk assessment methodology; Figure S2: PFOA equivalents (PFOAeq) calculated for each PFAS quantified by target analysis at 24 sampling locations (1–24); Figure S3: Average concentrations (ng/L) of semi-quantified PFAS compounds detected in surface waters of the five investigated rivers; Table S1: Native and internal PFAS included in targeted analysis; Table S2: Description of sample points locations; Table S3: Suspect screening analysis workflow design; Table S4: Persistence, bioaccumulation, ecotoxicological effects, and human health effects of PFAS used to calculated the ToxPi scores; Table S5: PFAS compounds quantified in the targeted analysis; Table S6: PFAS compounds quantified in the targeted and SSA analysis; Table S7: Comparative analysis of maximum concentrations (ng/L) of targeted PFAS in European surface waters; Table S8: SSA identified PFAS and their main properties; Table S9: Summary of persistence (P), bioaccumulation (B), and toxicity (T) indicators for investigated PFAS, with calculated ToxPi and risk index (RI) values.
