# Supplementary material for: Integrated Targeted and Suspect Screening Workflow for Identifying PFAS of Concern in Urban-Impacted Serbian Rivers
Source: Toxics. 2026 Jan 14;14(1):78. doi: 10.3390/toxics14010078 (PMC12845660; doi:10.3390/toxics14010078)
Supplement: Supplementary file 1 [file toxics-14-00078-s001.zip › Supplementary material.pdf]

Integrated targeted and suspect screening workflow for identifying PFAS of concern in urban-impacted Serbian rivers

Igor Antić[1†], Maja Buljovčić[1†], Richard E. Cochran[2], Jelena Živančev[1], Marta Llorca[3], Marinella Farré[3], Dušan Rakić[1], Ralf Tautenhahn[4], Nataša Đurišić-Mladenović[1],*

[1] University of Novi Sad, Faculty of Technology Novi Sad, Bulevar cara Lazara 1, 21000 Novi Sad, Serbia,

[2]Thermo Fisher Scientific, Bannockburn, IL

[3] Environmental and Water Chemistry for Human Health (ONHEALTH), Institute of Environmental Assessment and Water Research (IDAEA-CSIC), c/Jordi Girona 18-26, 08034, Barcelona, Spain

[4]Thermo Fisher Scientific, San Jose, CA

*corresponding author: natasa.mladenovic@uns.ac.rs, natasadjm@tf.uns.ac.rs

†These authors contributed equally to this work

SUPPLEMENTARY MATERIAL

Text S1. Detailed description of sample locations

This supplementary section provides a detailed description of all sampling sites included in the study. It outlines the hydrological characteristics, geographical setting, and main anthropogenic pressures relevant to each water body. The monitoring campaign encompassed major surface water systems of northern Serbia, the Danube, Sava, Tisa, and Tamiš Rivers, as well as the Great Bačka Canal, the principal component of the Danube–Tisa–Danube (DTD) canal network. For each system, the sampling locations are presented together with their approximate coordinates, dominant land use, and known sources of pollution, including municipal wastewater, industrial effluents, and agricultural runoff. These details provide the environmental and hydrological context necessary to interpret PFAS occurrence patterns, discussed in the main manuscript. The Danube River, Serbia's longest river with a total length of approximately 588 km within national borders, receives input from numerous tributaries and flows through diverse landscapes, including urban, agricultural, and industrial zones. As such, it is subject to multiple contamination sources, particularly wastewater discharge and industrial effluents from sources such as petrochemical, metallurgical, and textile industries. Higher contamination levels were anticipated near urban and industrial centers such as Bačka Palanka, Novi Sad, Belgrade, Smederevo, and Zemun (samples 3, 4, 13, 14, 15, 16, 17, 18, 23, and 24, respectively). The Great Bačka Canal was sampled at locations of Kula, Vrbas, and Srbobran (samples 5–10, respectively). The Great Bačka Canal, stretching over 118 kilometers, is a major component of the Danube–Tisa–Danube (DTD) canal system, a complex hydrotechnical network located in the Vojvodina region of northern Serbia. The DTD system consists of approximately 929 kilometers of interconnected canals, rivers, and regulatory structures designed for multi-purpose water management, including flood control, irrigation, drainage, navigation, and environmental protection. The Great Bačka Canal connects the Danube River near Bezdan with the Tisa River near Bečej, traversing a densely populated and agriculturally significant area. Its hydrological and ecological condition is of particular interest due to its historical role as an industrial wastewater recipient and its inclusion in various national and transboundary water quality monitoring programs. The Tisa River, which flows through Serbia for about 164 km, is an important tributary of the Danube and supports agriculture, water supply, and biodiversity in northern Serbia. Sampling was performed near Bečej (samples 11 and 12). In Serbia, the Tisa River flows through several urban settlements before reaching the town of Bečej, including Kanjiža, Senta, Ada, and Novi Bečej. These municipalities are situated along the central flow of the river and represent a mixture of urban, agricultural, and industrial zones. Some of them, such as Senta and Kanjiža, serve as regional industrial centers, with a long-standing history of textile, chemical, and food production. The river corridor in this section of the Tisa basin is also characterized by intensive agricultural land use, wastewater discharge points, and water abstraction for irrigation, all of which contribute to the river's chemical and ecological status prior to its confluence with the Great Bačka Canal in Bečej. The Tamiš River (140 km within Serbia), a transboundary watercourse originating in the Carpathian Mountains of Romania, enters Serbia near the village of Jaša Tomić and flows southward through the Banat region, eventually joining the Danube River near Pančevo. Along its course in Serbia, the Tamiš passes through or near several urban and rural settlements, including Jaša Tomić, Sečanj, Botoš, and Pančevo. This section of the river basin is characterized by a combination of intensive agriculture, livestock farming, and industrial activity, particularly near Pančevo, which is one of the major petrochemical and industrial hubs in the region. These anthropogenic pressures, coupled with inputs from municipal wastewater discharges and diffuse agricultural sources, make the Tamiš an important river system for studying the occurrence and

transport of CECs. The samples were taken in Pančevo (samples 19 and 20). The Sava River, one of the major tributaries of the Danube River, flows through four countries in Southeast Europe before entering Serbia near Jamena, in the northwest. Within Serbia, the Sava passes through or near several important urban and industrial centers, most notably the capital city, Belgrade, where it joins the Danube. Other Serbian settlements along the Sava include Šabac and Sremska Mitrovica, both of which contribute to the river's anthropogenic load through municipal wastewater, industrial effluents, and agricultural runoff. The river's catchment area in Serbia is characterized by a mix of dense urbanization, intensive agriculture, and industrial zones, making the Sava a complex system in terms of both ecology and chemistry. Due to these pressures, the Sava River is frequently included in national and international monitoring programs targeting CECs, including PFAS, pharmaceuticals, and personal care products, which may pose risks to both aquatic ecosystems and drinking water resources. The Sava River was sampled near New Belgrade (samples 21 and 22), a densely populated and commercial district on the left bank of the river.

Criteria	Attributes	Unit	Scaling	Weight	Software	Model(s)
Persistence (P)	Biowin1<br>Biowin3<br>Biowin5	unitless<br>unitless<br>unitless	x<br>x<br>x	1/9<br>1/9<br>1/9	EPI Suite v4.11	BIOWIN v4.10<br>BIOWIN v4.10<br>BIOWIN v4.10
Bioaccumulation (B)	BCF<br>Log Kow	L/kg<br>unitless	log 10(x)<br>x	1/6<br>1/6	EPI Suite v4.11	BCFBAF v3.01<br>KOWWIN v1.68
Toxicity (T): Ecotoxicological effects	Fish LC50 (96 h)<br>Daphnid LC50 (48 h)<br>Green Algae EC50 (96 h)	mg/L<br>mg/L<br>mg/L	-log 10(x)<br>-log 10(x)<br>-log 10(x)	1/18<br>1/18<br>1/18	ECOSAR v2.0	ECOSAR v2.0<br>ECOSAR v2.0<br>ECOSAR v2.0
Toxicity (T): Human health effects	Carcinogenicity	Unitless	x	1/60	VEGA v1.2.0	CAESAR v2.1.10<br>ISS v 1.0.3<br>IRFMN-ISSCAN-CGX v1.0.1<br>IRFMN-Antares v1.0.1
	Developmental toxicity	unitless	x	1/60	VEGA v1.2.0<br>VEGA v1.2.0<br>T.E.S.T. v5.1.1<br>QSAR Toolbox v 4.5	CAESAR v2.1.8<br>PG v1.1.2<br>Consensus<br>DART
	Mutagenicity	unitless	x	1/60	VEGA v1.2.0	Consensus v1.0.4<br>CORAL v1.0.1<br>IRFMN-VERMEER v1.0.1<br>IRFMN v1.0.2
	Endocrine disrupting effect	unitless	x	1/60	VEGA v1.2.0	IRFMN-CERAPP v1.01<br>IRFMN-COMPARA v1.0.1<br>IRFMN v1.0.0
	Hepatotoxicity	unitless	x	1/60	VEGA v1.2.0	IRFMN v1.0.1
	Skin irritation/corrosion	unitless	x	1/60	QSAR Toolbox v 4.5	BfR
	Eye irritation/corrosion				QSAR Toolbox v 4.5	BfR
	Skin sensitization	unitless<br>unitless	x<br>x	1/60<br>1/60	VEGA v1.2.0	CAESAR v2.1.7<br>IRFMN-JRC v1.0.1<br>NCSTOX v1.0.1<br>TOXTREE v1.0.0
	Repeated dose toxicity	mg/kg	-log 10(x)	1/60	VEGA v1.2.0	IRFMN-CORAL v1.0.1
	Oral rat LD50	mg/L	-log 10(x)	1/60	T.E.S.T. v5.1.1	Consensus

*Physicochemical and toxicological parameters were obtained from predictive models within the U.S. EPA Estimation Programs Interface Suite (EPI Suite), Ecological Structure Activity Relationships model (ECOSAR), and Toxicity Estimation Software Tool (T.E.S.T.), as well as from the OECD QSAR Toolbox and VEGA platform (Istituto di Ricerche Farmacologiche Mario Negri IRCCS). The octanol–water partition coefficient (log K_{ow}), bioconcentration factor (BCF), and biodegradability indices (BIOWIN 1, 3, and 5) were used to assess persistence and bioaccumulation potential. BIOWIN 1 estimates the probability of rapid biodegradation; BIOWIN 3 predicts the time required for ultimate biodegradation under aerobic conditions; BIOWIN 5 assesses ready biodegradability based on the Japanese MITI test. Repeated dose toxicity was expressed as the no-observed-adverse-effect level (NOAEL), defined as the highest exposure level with no biologically significant adverse effects. Scaling and weighting procedures were applied to harmonize the data from different models before ToxPi score calculation.

Figure S1. Risk assessment methodology

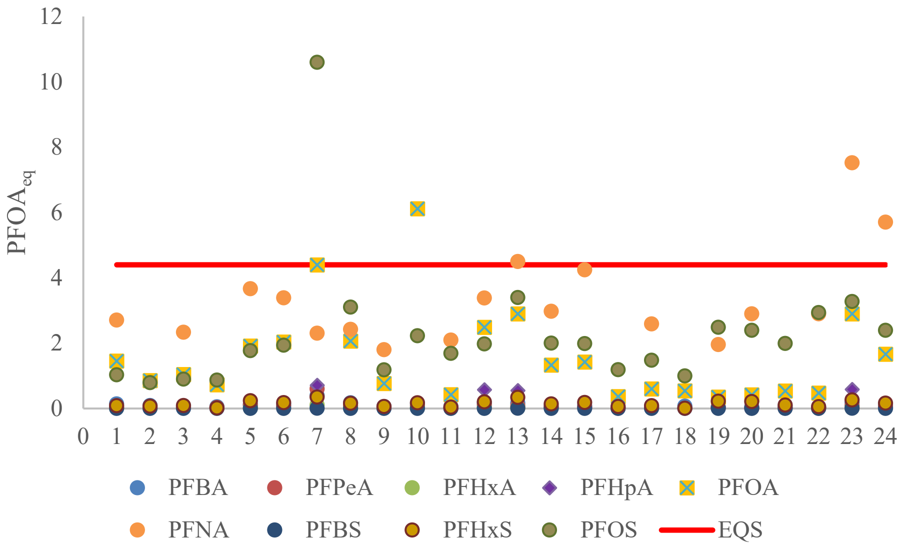

Figure S2. PFOA equivalents ($PFOA_{eq}$) calculated for each PFAS quantified by targeted analysis of surface waters from 24 sampling locations (1-24); the red line represents the proposed Environmental Standard Quality for surface waters (4.4 ng/L as PFOA equivalents).

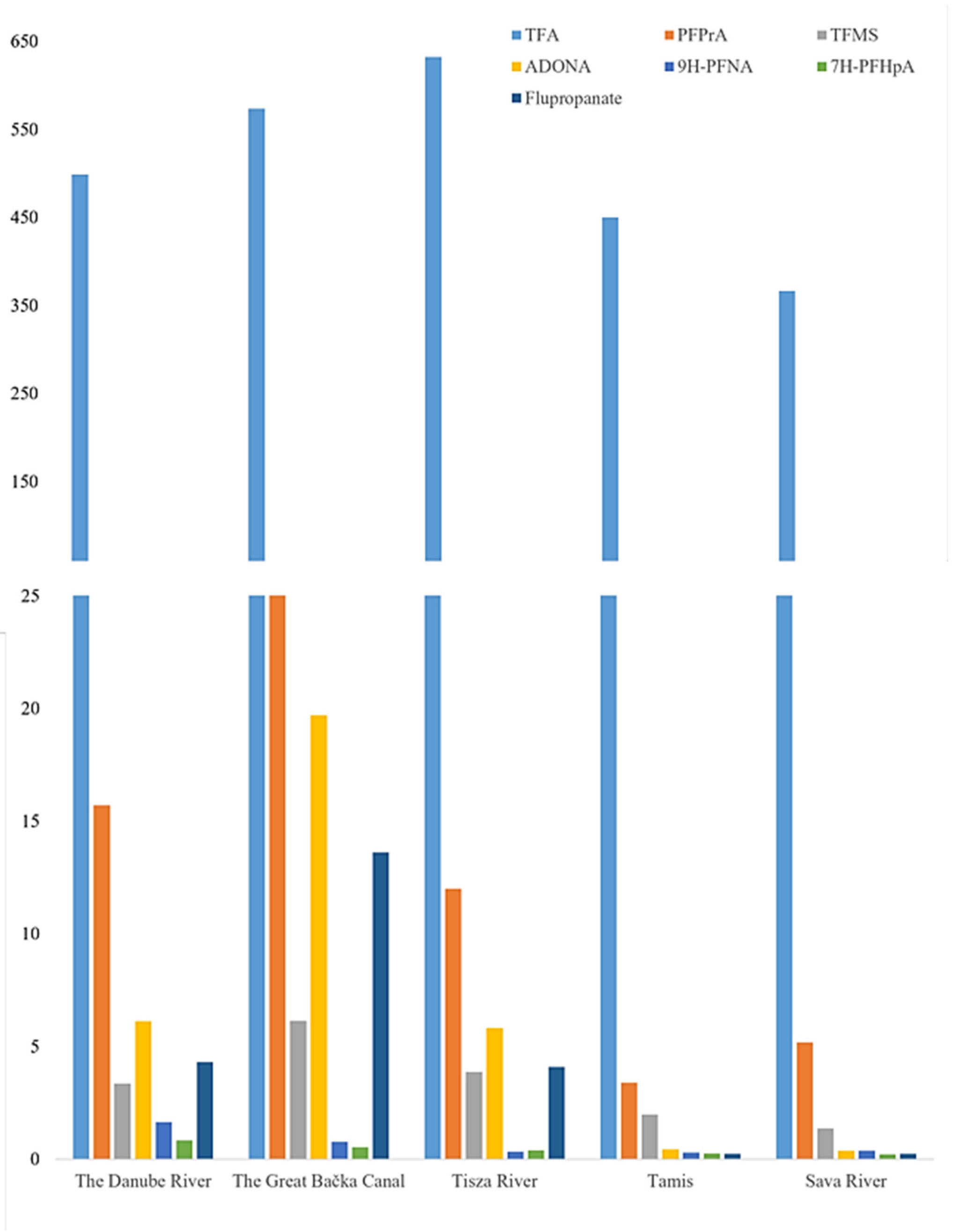

Figure S3. Average concentrations (ng/L) of semi-quantified PFAS compounds detected in surface waters of the five investigated rivers.

Trifluoroacetic acid (TFA), 4,8-Dioxa-3H-perfluorononanoic acid (ADONA), perfluoropropionic acid (PFPrA), 9H-Hexadecafluorononanoic acid (9H-PFNA), trifluoromethanesulfonic acid (TFMS), and 7H-Perfluoroheptanoic acid (7H-PFHpA).
